# Technological aids for improving longitudinal research on substance use disorders

**DOI:** 10.1186/s12913-016-1630-0

**Published:** 2016-08-10

**Authors:** David Farabee, Marya Schulte, Rachel Gonzales, Christine E. Grella

**Affiliations:** Semel Institute for Neuroscience and Human Behavior, David Geffen School of Medicine, University of California at Los Angeles, 11075 Santa Monica Blvd, Suite 200, Los Angeles, CA 90025 USA

## Abstract

**Background:**

There is a broad consensus that addictive behaviors tend to be chronic and relapsing. But for field studies of substance users, successfully tracking, locating, and following up with a representative sample of subjects is a challenge.

**Methods:**

The purpose of this paper is to provide a general overview of how current technological aids can support and improve the quality of longitudinal research on substance use disorders. The review is grouped into four domains: (1) tracking and locating, (2) prompting/engaging, (3) incentivizing, and (4) collecting data.

**Results & conclusions:**

Although the technologies described in this review will be modified or replaced over time, our findings suggest that incorporating some or all of these currently available approaches may improve research efficiency, follow-up rates, and data quality.

## Background

The past 20 years have seen substantial changes in our understanding of substance use disorder (SUD). It is no longer viewed as deviant behavior committed by the morally weak; nor is a single, episodic intervention considered to be effective treatment [1]. SUD is considered to be a chronic problem, associated with high rates of morbidity, mortality, and other adverse conditions [2]. This emerging recognition has underscored the importance of studying the epidemiology, natural history, and treatment of substance use disorders over the long term. Indeed, as former director of the National Institute on Drug Abuse (NIDA), Alan Leshner, noted, “…we will make progress in dealing with drug issues only when our national discourse and our strategies are as complex and comprehensive as the problem itself” [3].

However, capturing the complexity of SUD over time requires high rates of study sample retention. This can be a challenge with individuals who have unstable living conditions and/or frequent criminal justice involvement. One meta-analysis of 85 longitudinal studies of substance abuse clients found that nearly one-third of subjects were lost to attrition within 36 months [4]. A more recent survey of NIDA-funded investigators engaged in longitudinal research found that 27 % of the studies fell below the 80 % guideline for program follow-up rates [5, 6]. Although there is a lack of evidence for a strict cut off for research findings, this poses a significant threat to the validity of findings in the SUD literature, as follow-up rates below 80 % have been shown to produce dramatically biased estimates of drug use and related behaviors [7]. For example, participants who remain in longitudinal studies of SUD treatment outcomes differ from study dropouts/non-responders in levels of treatment participation, use of other drugs, and level of education [8, 9].

Fortunately, a number of technological advances have emerged—or become sufficiently commonplace—that improve researchers’ ability to maintain high follow-up rates in longitudinal studies. This paper summarizes some of these technological aids with regard to their ability to locate, engage, and incentivize research participants over time and facilitate data collection.

### Locating

The retention of subjects at adequate levels is necessary both to maintain statistical power and to reduce attrition bias in resultant follow-up samples. Subject retention is particularly challenging when study samples are drawn from populations characterized by residential instability, contact with the criminal justice system, and problems in psychosocial functioning, as is often the case in stu dies of individuals with SUDs. Inadequate sample size due to attrition has long been documented in review and meta-analytic publications as a threat to power, impacting the internal and external validity of longitudinal studies (e.g., [10–12]). Hence, well-established methods have been developed and refined to enhance subject retention in longitudinal studies of substance users and other vulnerable populations [13–17] to increase their participation in ongoing follow-up interviews [18, 19]; and to address human subject concerns regarding privacy and confidentiality of study participants [20]. Standard procedures utilized in longitudinal research include collection of detailed personal information from participants at study intake, including obtaining participants’ consent to contact family members and others who may be able to provide information on their whereabouts; frequently contacting study participants (by mail or phone) to maintain rapport, update locator information, remind participants of scheduled interviews, and enhance their connection to the study; using monetary or non-cash incentives to increase willingness to participate in ongoing interviews [13, 18, 21]; and using publicly available records, Internet-based search engines, or subscription-based databases (e.g., LexisNexus) to track individuals who have lost contact with the study. When resources have permitted it, some studies have sent interviewers into the field to track subjects at local hangouts or public places, hoping to find hard-to-locate participants and induce their participation through face-to-face contact [14].

Given the large body of research with substance users, methods for tracking, locating, and retaining them in longitudinal studies have become increasingly more sophisticated over time, including the use of empirically based methods that enhance follow-up rates. For example, several studies have examined the characteristics of individuals associated with study sample attrition [22–25] the optimal amount and schedule of remuneration to maximize retention [18, 26] and the relationship of number of follow-up attempts with study retention and participant outcomes [27–32]. As a result, follow-up methods have been tailored for specific subpopulations of participants recruited from different study sites (e.g., hospital emergency rooms, general medical clinics, specialty clinics) or types of participants (e.g., adolescents, individuals with co-occurring disorders). Efforts to systematically evaluate the effectiveness of follow-up efforts have led to the conclusion that the use of multiple strategies (e.g., telephone, database searches, mailed reminders), the scaled use of resources to target the hardest-to-locate participants with more intensive methods, the use of schedules of increasing subject payment, flexibility in scheduling field interviews, and persistence are required to maximize follow-up rates [31, 33].

Paralleling recent developments in intervention research that have used mHealth or Internet-based strategies to deliver treatment or continuing care, methods for tracking and retaining study participants in longitudinal research are similarly incorporating new technologies to enhance subject retention. As investigators increasingly incorporate these methods in follow-up research, new issues and challenges have arisen. We briefly review these issues with regard to: (1) use of cell phones, (2) use of social networking sites, and (3) use of Internet databases for tracking and locating.

#### Cell phones

Cell phones have become an important means for delivering treatment and continuing care interventions for substance-using populations, both through text messaging and smartphone applications [34]. An initial concern regarding the incorporation of cell phones within research and follow-up efforts has been whether substance-using participants, particularly those who are impoverished and/or homeless, have access to them. In formative survey research for developing a mobile phone intervention to sustain recovery among women offenders, Scott and colleagues [35] found that 83 % of the women had cell phones and 30 % of those were smartphones. Reported comfort level with use of cell phones was high, although many had access only through prepaid minutes, rather than through extended plans. In a survey and semi-structured interviews conducted with research staff from 10 sites participating in the NIDA Clinical Trials Network, Mitchell and colleagues [36] found that interviewers perceived cell phones to be especially advantageous for retaining contact with participants who were residentially unstable and did not have landlines.

#### Social networking sites

Social networking sites provide an opportunity to engage study participants by establishing a study profile, which can utilize study logos and other ways to “brand” the study. Researchers are able to conduct *Facebook* searches and send private emails to study participants who are located. As with cell phones, questions about the extent of involvement of study participants in social networking pertain to its potential utility for maintaining contact with study participants. The utility of these approaches may be limited with older generations who are not active users of social networking, as was found in a study that used social networking sites (*Facebook* and *Friends Reunited*) to locate participants in studies of childhood behavior from decades earlier [37]. However, researchers participating in the NIDA CTN found that many homeless individuals routinely had access to the Internet through public libraries, and were able to send and receive emails to study investigators [36].

Despite the potential limitations in access for some study participants, several studies have successfully used social networking to locate respondents, including in a longitudinal study of methamphetamine users [38] and a study of children involved in social welfare services [39]. One study found *Facebook* to be effective in locating hard-to-reach participants who had participated in an intervention program for children in at-risk families, reducing study attrition by 16 % [40]. Further, the investigators found some differences in characteristics of the children located via their mothers’ *Facebook* site, including ethnicity (more likely to be White or Aboriginal than recent immigrants) and language receptivity, which was higher among those located through *Facebook*.

#### Internet for tracking and locating

Although use of public records has long been a tool for tracking and locating respondents, Internet access through various search engines has vastly expanded access to the broad range of information that is available online. However, there is also increasing attention to privacy concerns, particularly related to protected health information and other confidential information. Recent security breaches that have targeted commercial vendors, government databases, and health providers have heightened concerns about the ramifications of access to information in ways that may pose harm to individuals.

Freely available Internet tools include web search engines, such as Google, Yahoo, People Search, and Bing, among others; telephone directories, including reverse directories that use telephone numbers to generate addresses; criminal justice records from county jails, state Departments of Corrections, or the Federal Bureau of Prisons; death records obtained through online obituaries, state Vital Records, or the National Death Index; and subscription-based people-finder services, such as Checkmate and People Smart. Access to some sites, such as the Social Security Death Index, requires date of birth and use of the participant’s social security number, and also require a fee. Several studies have closely tracked their experiences with these various databases and methods, and compiled useful recommendations for ways to maximize their application [36, 41, 42].

A study’s use of these search engines needs to be clearly communicated to study participants at the time of intake, including solicitation of their consent for their use. Upon study intake, researchers may benefit from initiating these strategies, such as conducting Internet-based searches and developing a study social networking site, in order to maintain subject contact and up-do-date locator information. This was evident in a study conducted in Canada, in which use of Internet-based searches to obtain telephone numbers at the time of study intake enhanced the researchers’ ability to locate participants who had subsequently moved and/or were using different names after 6 to 8 years [43].

### Engaging

Maintaining participant engagement, particularly when there may be long periods between assessment points, is essential to study retention and ensuring the validity of the results. Clinical populations, in particular, face significant barriers in terms of retention; frequent change in residency and generally high mobility make follow-up a challenge [44]. The use of multiple methods for proactive retention not only prevents attrition but can actually improve follow-up rates [45]. Communication technologies, in particular, are increasingly becoming important tools that researchers can use to interact with participants and promote study involvement.

According to a recent Pew Research Center poll [46], approximately 90 % of American adults have a cellphone and nearly two-thirds have a smartphone [47]. Populations with traditionally very limited or no Internet access, such as lower socioeconomic and racial/ethnic minority groups, are able now to use their phone for connecting to online information [47]. Thus, there is greater ability to engage and prompt large and diverse samples in order to improve study retention. Email invitations can serve as reminders for upcoming follow-up appointments as well as provide participants links to complete Internet-based follow-up assessments. In a qualitative study examining participant preferences in health services research, Hunter, Corcoran, Leeder, and Phelps [48] found that neither age nor gender affected participants’ preference for email invitations and web-based surveys. The authors, however, presented a wide range of responses regarding the point at which participants would feel “spammed” or simply ignore and delete email prompts; some stated every one to two weeks was acceptable while others felt monthly reminders would be excessive. Therefore, it may be helpful to note at intake participant concerns about spamming in order to reap the benefit of email reminders without over-prompting to annoyance.

Similar to email, the ubiquity of cellphones has made text messaging an easy and low-cost method for staying connected to participants in order to increase long-term follow-up rates [49]. Several studies have now shown text messaging to be a useful method for assessment and intervention delivery (see *Measuring Drug Use and Related Outcomes* section); however, it also provides a simple way to send appointment reminders for telephone and face-to-face interviews. Additionally, text messaging is useful for sending thank-you notices for participation and acknowledging important dates, such as birthdays and milestones of study participation (e.g., one-year participation anniversary). Regular engagement with study staff has been shown to aid in retention [44, 50, 51] and personalized text-based interactions with staff may be valuable in increasing personal investment and identification with the project.

While phone numbers and email addresses are vulnerable to change, social media (e.g., Facebook, Twitter) usernames are generally stable. Facebook is becoming a more widely used tool for tracking and locating hard-to-find participants (see *Locating* section); however, gaining IRB approval for collecting social media contact information can be a cumbersome process [36, 38] . To maximize time allowed within the study to use Facebook (or other social media), as well as reduce staff time spent searching and confirming participant identities, it is recommended that researchers navigate and resolve IRB issues at the start of the study. Kim, Hickman, Gali, Orozco, and Prochaska [45] tracked substance use and mental health functioning in a high-risk, low-income population with serious mental illness at 3-, 6-, and 12-months post-baseline. They found that follow-up rates can actually increase over time when multiple strategies for maintaining contact are implemented. Similar findings were noted in a recent study examining recruitment and retention efforts with a veteran population with substance use disorders; the collection of social network usernames along with traditional contact information at baseline assisted in reducing loss to follow-up [52].

### Incentivizing

There is growing evidence supporting the ease and effectiveness of issuing subject payments electronically rather than by vouchers, money orders, or even cash. Some of the initial concerns surrounding debit-card payments for research participants have been addressed by vendors (e.g., Greenphire, CT Payer) specializing in issuing payments for clinical trial participants.^1^

The availability of electronic payment methods has expanded rapidly over the last 20 years, along with improvements in the ease of their use and security of payments. According to the Federal Reserve System [53], the number of payments (in the United States) issued by debit cards exceeded the number of credit card payments for the first time around 2004. Likewise, the number of prepaid card payments also increased—by more than 3 billion from 2009 to 2012. The use of mobile wallets also has increased sharply. This category refers to payments using the cell phone short message service (SMS), a mobile application, a virtual cloud based account, or near field radio-frequency identification (RFID) linked to a mobile device. In 2012, more than 250.6 million mobile payments were made using a mobile wallet application. In contrast, the number of checks paid has declined by half in the last decade alone.

The reason for the widespread adoption of this technology is clear: relative to the use of checks—or even cash in some contexts—debit cards offer a faster, simpler way of making financial transactions. Though virtually everyone can appreciate the immediacy of electronic payments relative to checks or money orders, there is evidence that increased payment efficiency holds disproportionate appeal to substance abusers, due to their truncated sense of time and tendency to discount the value of delayed rewards at a rate of 2 to 4 times that of non-substance abusers [54]. Consequently, the value of immediate reinforcement appears to be especially acute for this population, underscoring the potential value of instantly creditable payment methods in longitudinal research with substance abusers.

Although the use of reloadable debit cards is slowly expanding in longitudinal substance use research, few researchers have published results from methodological studies comparing the effects of electronic payment methods on study engagement, follow-up rates, and staff time devoted to both paying and locating subjects over time. Our review of the literature revealed only one such study, which examined the use of debit cards as a means of enhancing follow-up rates in a longitudinal study of homeless drug users. In this study, De Jarlais, Perlis, and Settembrino [55] issued debit cards to a sample of 139 “urban nomads” and made deposits to their accounts each time they completed a telephone interview. The authors found that subjects with debit cards had substantially higher follow-up rates than similar subjects in two related studies that relied on traditional methods of issuing subject payments (81 % vs. 31 % and 67 %, respectively, at 6 months; 71 % vs. 10 % and 41 %, respectively, at 12 months).

In a more recent study, Farabee et al. [56] randomized 303 patients receiving SUD treatment into one of two incentivizing methods—money orders (MO) or a rechargeable incentive card (RIC). Participants were asked to call the researchers at the beginning of each calendar month for the ensuing 5 months in order to update their locator information—even if nothing had changed. Each call resulted in a $10 payment, issued immediately via the RIC system or by money order by mail. Research staff then located and interviewed all participants at Month 6. Contact logs assessed level of effort required to locate participants and conduct the follow-up interview. Relative to controls, RIC participants, especially those with low ability to defer gratification, initiated more monthly calls. Among outpatients, RIC participants made 39 % of the possible calls, whereas control subjects made 27 % (*p* < .01). Six-month follow-up rates (initiated by research staff) did not differ between RIC and control subjects (77 % overall). However, there were significant staff time savings in executing payments for those in the RIC condition.

With regard to ease of use, Farabee et al. [56] also assessed RIC users’ perception of reloadable debit cards and satisfaction with the process. They found that 98 % activated their cards. When asked if they had experienced any difficulties, 92 % reported that they had not, 6 % indicated that they had, and 2 % had not yet used their cards. When asked to compare the RIC method to other potential payment methods, RIC participants had a decided preference for the RIC method over grocery cards, money orders, and points redeemable for goods or services. Interestingly, there was even a slight, non-significant preference for the RIC method over cash payments.

Commercial vendors of rechargeable debit card payment methods for research participants (e.g., Greenphire, CT Payer) also provide user-friendly management information systems that simplify record-keeping and send automated reminders to both the researcher and participants of upcoming appointments. Moreover, these vendors are able to create limited-use bank accounts for individual study participants without having to access to the participants’ names or the nature of the study in which they are participating.

### Measuring drug use and related outcomes

Technology has played a role in widening the assessment of substance use and related factors in naturally occurring environments using a variety of media applications, including telephones or web-based electronic systems. Ecological Momentary Assessment (EMA), also called experience sampling, is a common technology/electronic-based data capture method used in substance abuse research. Specifically, EMA can collect current or very recent states of behavioral, emotional, or cognitive metrics in real-world environments either using event-based, time-based, or randomly prompted measurement models [57]. EMA data can be collected using random prompts, enabling assessment of the base rates of exposure to current or recent behavioral/emotional/cognitive states (i.e., craving/relapse triggers). In addition, EMA data can be captured using a participant-initiated approach, whereby participants are instructed to initiate an EMA entry immediately after a behavioral/emotional/cognitive state (i.e., after substance use, a craving, or a stressor occurred) [58, 59].

EMA data capture of behavioral, emotional, or cognitive indices has traditionally been collected using participant self-report; however, data capture has expanded to also include a combination of self-report with other sensory measurement (i.e., biological, physical, or auditory indices) programmed or linked to the electronic device. Specifically, self-reports entail participants completing ratings in response to prompts emitted by the device (with entries electronically time-stamped), whereas sensory data capture requires participants to use the electronic device to record pre-set sensory information (biological, auditory, or physical measures), such as heart-rate/pulse, voice, or environmental location-GPS, and number of steps/distance [60]. Examples of EMA studies collecting retrospective self-report include Project CHESS (Comprehensive Health Enhancement Support System [61] and Project ESQYIR (Educating and Supporting Inquisitive Youth in Recovery) [62]. In these studies, participants in recovery from substance use disorders received message prompts daily/weekly through a mobile phone platform (texting or mobile application) asking about substance use related behaviors, emotions, and cognitions. Other studies have focused on real-time self-initiated entries using random prompting. For example, Shiffman et al. [63] and Carter et al. [64] had participants carry PDA-based diaries on which they recorded their substance use behaviors when the PDAs “beeped” during different times of the day. Examples of EMA substance abuse studies using self-report in combination with sensory data collection include research by Mitchell et al. [65] and Epstein et al. [59] that used self-report EMA methods to examine real-time substance use behaviors, emotions (mood), cognitions (cravings), and sensory data (GPS location) to capture physical (neighborhood) metrics. The capture of both types of data occurred at randomly prompted times during waking hours.

A wide array of EMA methods have been used by researchers, providing a rich source of real-time, real-world observations. A common goal among the studies using EMA data collection methods is to obtain a complete or more holistic understanding of an individual’s recovery status (including an array of behavioral, emotional, cognitive, or environmental indicators, either via self-report or sensors) as he or she goes about daily life (during treatment or posttreatment). Much of the variability and creativity lies in how the researchers organize and analyze the EMA data to address unique research questions. Collectively, there are several benefits to using EMA methods. Data can be collected in greater frequency and in greater volumes within a short timespan, usually within 1–5 minutes. Self-reported data collection is flexible in terms of the metrics used (frequency, amount, ratings, or open-ended responses) [66]. Utilizing data from built-in sensor methods is also advantageous, as it allows the investigator to have more information without adding a commensurate burden to researchers or participants. One limitation of self-reported substance use behaviors collected with EMA research is the limited access to biological urine sample data; hence, face-to-face data collection is also needed in EMA data capture studies. Gonzales et al., [63] for example, used a combined EMA and in-person approach (i.e., EMA data capture occurred daily in combination with monthly face-to-face in-person assessments) to aid in corroborating self-reported results on drug use.

Response rates for substance abuse related studies utilizing EMA appear to be promising, but show considerable variation. For example, with tobacco users, response rates for reporting of cigarette use has ranged from 22 % [67], to 50 % [68, 69], to up to 90 % [70]. Regarding compliance with adhering to the EMA data collection assessments, studies show that substance abusing populations are responsive, ranging from 75 to 98 % responded prompts [51, 58–60, 71, 72]. A complexity in EMA compliance is its variability. Litt et al. [73], for example, reported that individuals with alcohol use disorder discharged from a treatment program recorded much more drinking on EMA than on a later retrospective assessment, suggesting good compliance; however, they also found that individuals with alcohol use disorder only recorded a minority of their lapses and that they sometimes suspended recording for a few days after a lapse [74]. Macedo, Maker, Latimer, and McAuley [75] recommend making follow-up phone calls in response to unanswered text prompts, which, in their study, boosted response rates from 54.8–74.2 % to 91.5–99 % over 12 months. Hence, good participant management procedures can yield high compliance.

## Conclusions

Longitudinal research provides valuable information for understanding the initiation and course of substance use. Drug-using populations, however, can be particularly difficult to engage and retain in long-term studies [7, 18]. While there has long been evidence that increasing contact attempts boosts follow-up rates [14, 76], newer findings have shown that the utilization of multiple methods, including technology-based communication, to contact participants can significantly improve retention [45, 52]. As such, locator forms should collect social media contact information in addition to standard contact methods when possible and inform participants that study staff will be periodically contacting them via various methods between assessments for the purposes of keeping up-to-date contact information. Together, email, texting, social media, and electronic payment systems constitute a multi-pronged approach that can simultaneously reduce staff costs and improve follow-up rates in longitudinal SUD research.

## Abbreviations

EMA, Ecological Momentary Assessment; NIDA, National Institute on Drug Abuse; RFID, Near Field Radio-frequency Identification (RFID); RIC, rechargeable incentive card; SMS, short message service; SUD, substance use disorder

## Footnotes

1 Few studies have reported results using other electronic payments such as Chase Quick Pay or Amazon e-gift cards, another potential quick electronic payment method.
